# Differences in the Activities of Eight Enzymes from Ten Soil Fungi and Their Possible Influences on the Surface Structure, Functional Groups, and Element Composition of Soil Colloids

**DOI:** 10.1371/journal.pone.0111740

**Published:** 2014-11-14

**Authors:** Wenjie Wang, Yanhong Li, Huimei Wang, Yuangang Zu

**Affiliations:** The Key Laboratory of Forest Plant Ecology Ministry of Education, Harbin, Heilongjiang Province, P.R. China; NERC Centre for Ecology & Hydrology, United Kingdom

## Abstract

How soil fungi function in soil carbon and nutrient cycling is not well understood by using fungal enzymatic differences and their interactions with soil colloids. Eight extracellular enzymes, EEAs (chitinase, carboxymethyl cellulase, β-glucosidase, protease, acid phosphatase, polyphenol oxidase, laccase, and guaiacol oxidase) secreted by ten fungi were compared, and then the fungi that showed low and high enzymatic activity were co-cultured with soil colloids for the purpose of finding fungi-soil interactions. Some fungi (*Gomphidius rutilus*, *Russula integra, Pholiota adiposa*, and *Geastrum mammosum*) secreted 3–4 enzymes with weak activities, while others (*Cyathus striatus*, *Suillus granulate*, *Phallus impudicus, Collybia dryophila, Agaricus sylvicola,* and *Lactarius deliciosus*) could secret over 5 enzymes with high activities. The differences in these fungi contributed to the alterations of functional groups (stretching bands of O-H, N-H, C-H, C = O, COO- decreased by 11–60%, while P = O, C-O stretching, O-H bending and Si-O-Si stretching increased 9–22%), surface appearance (disappearance of adhesive organic materials), and elemental compositions (11–49% decreases in C1s) in soil colloids. Moreover, more evident changes were generally in high enzymatic fungi (*C. striatus)* compared with low enzymatic fungi (*G. rutilus*). Our findings indicate that inter-fungi differences in EEA types and activities might be responsible for physical and chemical changes in soil colloids (the most active component of soil matrix), highlighting the important roles of soil fungi in soil nutrient cycling and functional maintenance.

## Introduction

The large majority of the 80,000+ fungal species so far named and described are likely to occur in the soil environment at some stage in their life-cycle [1]. Soil fungi are vital in proper functioning of the ecosystem by helping in the degradation of dead matter, releasing vital nutrients and leading to soil carbon sequestration in boreal forests [2]. Fungal activity is greatest in decomposing leaves and wood, and tends to diminish in the later stages of decomposition when bacteria become more dominant. Fungi utilize both simple and complex molecules as foods by the secretion of a variety of extracellular enzymes (EEA), including protease, cellulase, β-glucosidase, and chitinase [3]–[5]. These EEAs degrade plant protein, cellulose, hemicellulose, starch, and animal compounds such as chitin. In soil, phosphatases, extracellularly secreted by plants and microorganisms, play a key role in the phosphorus cycle, allowing the formation of inorganic phosphorus, the only phosphate form taken up by plants and microorganisms [6]–[9]. Laccase and polyphenol oxidase are the ligninolytic enzymes involved in the degradation of lignin as well as various xenobiotic aromatic compounds [10]–[12]. Various EEAs, which can indicate biological equilibrium, fertility, quality, and biological soil status [13], participate in soil nutrient transformation, energy metabolism, and degradation of various compounds [14], [15]. Current knowledge of fungal diversity in soil has been characterized through various next-generation sequencing methods in many papers [16]–[18] or observations of fruiting bodies present in the variable environment [1]. However, relatively few of these studies have examined functional diversity. Differences in soil fungal communities that affect C,N,P cycling may be due to inter-species differences in EEA activities related with C,N and P metabolism [19]. The functional differences that affect soil C and nutrient cycling need investigational support via comparison of inter-species soil enzymes and their effect on soil C, N, and P. As a hotspot for global warming in the northern hemisphere, northeastern China, with its fertile black soil, is also abundant with diverse soil fungi [20], [21]. Identification of inter-species differences of EEAs secretions could define the roles of soil fungi in cycling of C, N, and P substrates in the global warming process [19].

Soil colloids generally with effective diameters of around 10 nm to 10 µm are the most active portion of the soil and largely determine the physical and chemical properties of the soil and are usually composed of organic and inorganic colloids with different chemical and physical reactivity [22]–[25]. In general, the interaction between enzymes from different soil fungi may accelerate soil organic matter (SOM) decomposition and the release of nutrients, such as N and P. This SOM decomposition in soil colloids may directly affect physical absorption of organic matters [25]. Various technologically advanced techniques, such as infrared spectroscopy, scanning electron microscopy (SEM), as well as energy dispersive X-ray microanalysis (EDX), were employed to visually assess persistent fly ash constituents and quantitatively estimate functional group traits and elemental composition in soil colloids after interacting with soil fungi [25]–[27]. Whether the enzymatic secretion could affect soil colloid structure can be evaluated by co-culture of different fungi and soil particles. Selecting soil colloid, liquid, and solid co-culture between representative fungi and soil can be used to explore how the differences in enzymes influence the colloid surface structure and composition of soil colloid. These methodological advances could help clarify the influence of fungi and EEAs on the surface structure and composition of soil colloids [25], [27].

This paper puts forward the following hypothesis: EEA types and activities secreted by different fungi differentially regulate C, N, and P cycling, and such enzymatic differences should be one basis for the alteration of functional groups traits, surface images, and elemental composition in soil colloids during their co-cultures. After comparisons of the activities of 8 EEAs secreted by 10 fungi, co-cultures between two typical fungi (low and high enzymatic activities) and soil were used to explore possible enzymatic impact on soil colloids surface structure and composition changes.

## Materials and Methods

### 1 Study Materials

The target fungi species included 10 species. Seven species from Agaricales (Russula integra, Suillus granulatus, Pholiota adiposa, Collybia dryophila, Agaricus sylvicola, Gomphidius rutilus, and Lactarius deliciosus), Phallales (1 species: Phallus impudicus), Lycoperdales (1 species: Geastrum mammosum), and Nidulariales (1 species: Cyathus striatus) were collected from northeastern China. All these species were morphologically identified by Prof. Cunti Xiang (a professor in the Forest College, Northeast Forestry University),who has published a text book for forest mushroom of China [21]. R. integra, S. granulatus, G. rutilus, and L. deliciosus are ectomycorrhizal fungi, and they widely grow on the ground of pine forests. P. adiposa often grows on the trunk of poplar, willow, and birch trees, and can cause trunk decay. P. impudicus, C. dryophila, C. striatus, and G. mammosum usually grow on debris and litter on the forest floor and under shrubs [21].

Soil sample for co-culture with fungi was collected from the dark brown soil (0–20 cm) in the Experimental Forest Farm of Northeast Forestry University in northeastern China (45°43′6″N, 126°37′54″E). In a 20 m×20 m plot of a larch plantation, four soil samples were excavated by a soil cup of size 100 cm^3^ and mixed as a composite sample. The composite sample was placed in a cloth bag and aired in a dry ventilated room until they reached constant weight. After roots in the soil sample were carefully picked out, the samples were ground with a wooden rolling pin and passed through a 2 mm soil sieve. The gravels in the sample were sieved out, then was smashed in a motor pulverizer for approximately 3 min and passed through a 0.25 mm soil sieve for future laboratory experiment. This experimental forest farm administratively belongs to Northeast Forestry University (the working unit of the authors), and collections of samples have get an authority permission from the university and no protected species were sampled in this paper.

### 2 Fungal culture conditions

The strains of all 10 fungi were grown in Melin–Norkrans medium (MMN): CaCl_2_, 0.05 g; NaCl, 0.025 g; KH_2_PO_4_, 0.5 g; (NH_4_)_2_HPO_4_, 0.25 g; MgSO_4_·7H2O, 0.15 g; FeCl_3_ (1%), 1.2 ml; thiamine HCl, 0.2 ml; malt extract, 3 g; glucose, 10 g; stock solution of micronutrients (contents per liter: H_3_BO_3_, 2.86 g; MnCl_2_, 1.81 g; ZnSO_4_, 0.22 g; CuSO_4_, 0.08 g; and NaMoO_4_, 0.02 g), 1 ml; ultrapure water, 1000 ml; and 15 g of agar for agar media. The pH of the media was adjusted to 5.45–5.55 before autoclaving (121°C, 0.1 Mpa, 20 min). The medium was dispersed aseptically in a 10 cm culture dish and stored in a 4°C refrigerator. The strain was inoculated on the solid-medium, training repeatedly until no bacteria were observed [28].

MMN liquid medium without agar and supplemented with 0.3 g·L^−1^ yeast extract as a substitute for malt extract was used for culture of different fungi. carboxymethylcellulose (CMC), colloidal chitin and glucose were supplemented in the basal medium for stimulating enzyme secretion and the same medium was used for different fungi and different enzymatic determination. Aliquots (200 ml) of each medium were dispensed into 250 ml Erlenmeyer flasks and autoclaved at 117°C for 20 min. Each flask was inoculated with fungal colonies from one of the 10 fungal strains, and then cultured on a shaking table. After 15 days, culture media were filtered using a 4-layer gauze, then centrifuged (10,000×g) at 4°C for 10 min and enzymatic activity was determined in the supernatants, except for acid phosphatase, which was measured in the mycelium extract. Acid phosphatase activity in mycelium extract in the stationary period of mycelium growth (>72 hours cultivation) may much lower than those in culture liquid [29], the results in this paper may underestimate the acid phosphatase activity. Three replicate flasks for each soil fungi were used for the enzymatic measurements.

### 3 Determination of 8 extracellular enzymatic activities

Chitinase activity (EC 3.2.1.14) was studied according to the method of [30]. Activity was determined by the amount of end product, N-acetylglucosamine (NAG), secreted from the reaction. The reaction mixture consisted of 0.6 ml of the fungal filtrate and 1 ml of 1% colloidal chitin in 0.05 M acetate buffer (pH 5.6). Following incubation at 37°C for 3 h, the reaction was stopped by adding 0.75 ml dinitrosalicylic (DNS) reagent (0.63% DNS, 0.5% phenol, 0.5% sodium bisulfide, and 2.14% NaOH) [31] followed by heating for 10 min. The solution was then centrifuged and 1 ml of 18.2% Rochelle salt was added. Extinction of the supernatant was measured spectrophotometrically at 530 nm. The reaction mixtures containing only boiled culture filtrates were used as blanks. The amount of NAG released was determined from a calibration curve. One unit of enzyme activity was defined as the amount of enzyme required to produce 1 µmol of NAG per hour [3], [32].

Carboxymethyl cellulase (EC 3.2.1.4) activity was determined by measuring the amount of reducing sugars released in the reaction mixture containing: 0.9 ml of 1% w/v cellulose sodium carboxymethyl (Sigma, St. Louis, MO, USA) solution (dissolved by 0.1 M acetate buffer, pH 4.6), and 0.1 ml of the fungal filtrate. The mixture was maintained at 50°C for 30 min. The reducing sugars in the fungal filtrate were determined colorimetrically using the methods of [33]. Glucose was used as the standard. Measurements were made in a spectrophotometer at 520 nm wavelength in the presence of the blank. The reaction mixtures containing heat-inactivated post-culture liquids (boiled for 5 min) were used as the blanks. The carboxymethyl cellulase activity was expressed in units defined as the quantity of enzyme required to form 1 µmol of glucose per hour, under assay conditions.

β-glucosidase (EC 3.2.1.21) activity level was determined by measuring the amount of *p*-nitrophenol released in the reaction mixtures containing 0.9 ml of 0.01 M acetate buffer (pH 5.5), 50 µl of 40 mM *p*-nitrophenyl-β-D-glucopyranoside (Sigma), and 50 µl of the fungal extract. The mixtures were maintained at 45°C for 30 min and after the addition of 3 ml of 1 M Na_2_CO_3_, the amount of *p*-nitrophenol released was measured spectrophotometrically at 400 nm. The reaction mixtures containing boiled culture filtrates were used as the blanks. The cellulolytic enzyme activities were expressed in units defined as the quantity of enzyme required to form 1 µmol of *p*-nitrophenol (β-glucosidases) per hour, under assay conditions [3].

Proteinase (EC 3.4.21-24) activity was studied according to the method of [34]. The reaction mixture contained 750 µl of 2% sulfanilamide azocasein (Sigma) in 0.2 M acetate buffer (pH 4.5) and 750 µl of the culture filtrate. Following incubation at 37°C for 4 h, the reaction was stopped by adding 1.6 ml of 7% HClO_4_. Subsequently, the mixtures were centrifuged at 10,000×*g* for 10 min at 4°C. A 4 ml aliquot of the supernatant was added to 450 µl of 10 N NaOH. After vigorous shaking, the absorbance at 440 nm was assumed the activity of proteinase. The reaction mixtures containing boiled culture filtrates were used as the blanks. A solution of the fungal proteinase XIII (Sigma), in acetate buffer, pH 4.5, served as the standard for proteinase activity. The activity of proteinases was assumed to be the amount of enzyme required to form 1 µmol of tyrosine per hour at 37°C under the experimental conditions [3].

Acid phosphatase (EC 3.1.3.1) was measured using 100 µl of 0.015 M *p*-nitrophenyl phosphate, 500 µl acetate buffer (pH 4.8), and 100 µl of the fungal extract. The reaction mixture was incubated in a thermoblock at 37°C for 1 h. After this time, the reaction was stopped using 0.2 M Na_2_CO_3_. The fungi were homogenized with 0.01 M acetate buffer (pH 4.8), 0.01 M EDTA, 0.01% Triton X and 2.5% PVP (Polyclar, Serva) [35]. The *p*-nitrophenol formed was determined spectrophotometrically at 400 nm. The reaction mixtures containing boiled culture filtrates were used as the blanks. One unit of acid phosphatase activity was defined as the amount of enzyme required to form 1 µmol of *p*-nitrophenol per hour [3].

Polyphenol oxidase (EC 1.14.18.1) activity was measured using catechol as a substrate [36]. In test tubes, 0.75 ml of the supernatant from the fungal cultures was combined with 2 ml 0.1 M citric acid-sodium citrate buffer (pH 5.5). Following the addition of 0.25 ml of catechol (or distilled water for controls), test tubes were vortexed and incubated at 30°C for 30 min, and then the final absorbance was determined (420 nm) with a spectrophotometer. The reaction mixtures containing boiled culture filtrates were used as the blanks. One unit of polyphenol oxidase activity was defined as the absorbance changed 0.01 unit per hour, at OD_420_.

Laccase (EC 1.10.3.2) activity was measured using 2, 2′-azinobis-3-ethylbenzothiazoline-6-sulfonic acid (ABTS) as a substrate. The reaction mixture contained 0.5 ml of 1 mM ABTS in 2.0 ml 0.1 M acetate buffer (pH 4.6) and 0.2 ml of the culture filtrate. Following incubation at 28°C for 30 min, and then the final absorbance was determined (600 nm) with a spectrophotometer. The reaction mixtures containing boiled culture filtrates were used as the blanks. One unit of laccase activity was defined as an absorbance change of 0.01 unit per hour, at OD_600_ [33].

Guaiacol oxidase (EC 1.11.1.7) activity was measured using guaiacol as a substrate. The reaction mixture contained 80 mM guaiacol in 3.0 ml of 0.1 M acetate buffer (pH 4.6), and 0.5 ml of the culture filtrate. Following incubation at 28°C for 30 min, the final absorbance was determined (490 nm) with a spectrophotometer. The reaction mixtures containing boiled culture filtrates were used as the blanks. One unit of guaiacol oxidase activity was defined as the absorbance changed of 0.01 unit per hour, at OD_490_ [33].

### 4 Co-culture of typical fungi in dark brown soil and preparation of soil colloids

In order to explore how enzymes influence soil colloid surface structure and composition, liquid and solid co-culture between two typical fungi, *C. striatus* and *G. rutilus,* and dark brown forest soil were carried out. These two fungi were selected based on their measured enzymatic activity (see section 2.3). *C. striatus* had relatively higher enzymatic activities, while *G. rutilus* had lower enzymatic activities and has been often studied previously [19].

Co-culture of the fungi and dark brown soil in liquid medium (liquid co-culture): aliquots (100 ml) of each medium were dispensed into 150 ml Erlenmeyer flasks. Five grams of dark brown soil was then added before autoclaving (121°C, 0.1 Mpa, 20 min). Each flask was inoculated with fungal colonies of *C. striatus* and *G. rutilus* and then cultured on a shaking table. The control (soil and liquid media) was not inoculated with any fungal colonies. After 21 days, the soil colloids were separated as follows. Firstly, the culture media were filtered using a 4-layer gauze. The suspension was allowed to stand undisturbed for more than 24 h. Sands and silts in the soil sample were gradually deposited at the bottom of the beaker, whereas the soil colloids were left in the suspension, indicated as a turbid solution. The upper suspension was carefully decanted to a centrifuge tube and then centrifuged at 12000 rpm for 10 min. The precipitates (10 mg) were dissolved with 10 ml of ultrapure water and regarded as the soil colloidal solution [23], [25].

Fungi inoculated on dark brown soil (Solid co-culture): 20 grams of air-dried soil was put into a canned bottle about 100 ml with 15 ml MMN liquid medium for submerging soils completely, and then autoclaved at 117°C for 20 min. Each bottle was inoculated with fungal colonies of *C. striatus*, *G. rutilus* and no fungi (as control) and then cultured on an incubator (25°C). After 3 months co-cultivation at room temperature, the air-dried soil after the removal of the fungal hyphae at surface was used for soil colloid separation as follows: 5 grams of air-dried soil was fully dispersed in 500 ml of ultrapure water in a 1000 ml beaker. The suspension was allowed to stand undisturbed for more than 24 h. The upper suspension was carefully decanted to a centrifuge tube and then centrifuged at 12000 rpm for 10 min. The precipitates (10 mg) were dissolved with 10 ml of ultrapure water and regarded as the soil colloidal solution.

For both liquid- and solid-cocluture, the soil together with the media was autoclaved for one time. Owing to the sterilization time could significantly affect soil microbiological properties and soil enzymatic activities [37], [38], our results may differ from those with more sterilization processes.

### 5 Observations of functional group changes in soil colloids

The soil colloidal solution was centrifuged at 12000 rpm for 10 min. After air-drying, the precipitates (1 parts) were diluted with KBr (100 parts) mixing powder and separately pressed to obtain self-supporting disks. Tablets for infrared (IR) spectrum measurements were prepared by pressing the powder mixture at a load of 8 tons for 8 min. The IR spectrum was obtained by a compact Fourier transform infrared spectrophotometer (IRAffinity-1, Shimadzu, Kyoto, Japan) and recorded across a wave number range of 4000–500 cm^−1^ at a resolution of 4 cm^−1^ [25].

Four functional groups were selected for IR analysis, as shown in Table 1: 3800–3000 cm^−1^ is the O-H stretching region of carboxylic acids, phenols, alcohols, N-H stretching of amines and amides, aromatic C-H stretching; O-H stretching of clay minerals and oxides as well as sorbed water. 3000–2820 cm^−1^ indicates aromatic and aliphatic C-H stretching; 1800–1485 cm^−1^ is C = O stretching of carboxylic acids, amides, and ketones, and asymmetric COO- stretching of carboxylic acid salts; 1350–820 cm^−1^ are P = O of inorganic phosphates, inorganic carbonates, C-O stretching, O-H bending of –COOH and C-O stretching of polysaccharides, bending of structural O-H and Si-O-Si stretching in inorganic clay minerals and oxides [39]. The area of absorption peaks, which could semi-quantitatively describe the content of functional groups, was calculated using Image J software (http://imagej.nih.gov/ij/) [25].

**Table 1:** Wave number ranges used for data analysis and their dominating chemical compounds and functional groups [39].

	Wave number(cm^−1^)	Attribution of IR spectra.	
		Dominating chemical compounds	Functional groups and bonds
I	3800–3000	Carboxylic acids, phenols, alcohols, amines, amides, aromatic series, clay minerals and oxides as well as sorbed water	O-H stretching, N-H stretching, aromatic C-H stretching
II	3000–2820	Aliphatic series	Aliphatic C-H stretching
III	1800–1485	Carboxylic acids, amides, ketones, salts of carboxylic acids.	C = O stretching, asymmetric COO- stretching
IV	1350–820	Inorganic phosphates and carbonates, polysaccharides, clay minerals and oxides	P = O, C-O stretching, O-H bending, Si-O-Si stretching

### 6 Observation of surface structure and elements composition changes in soil colloids

The soil colloidal solution (1–2 drops) was dropped on the sample stage and then air-dried before image observation using a scanning electrical microscope and analysis of element composition changes via an Energy-dispersive X-ray spectroscopy (EDX) (Quanta200, FEI, USA). The samples were sputter-coated with a thin layer of gold-palladium (5–10 nm, 25 mA, 3 min) at room temperature by using a sputter coater before the examination. Detail of the method can be found in [25], [27].

### 7 Data Analysis

Analysis of variance (ANOVA) and post hoc multiple comparisons between the levels of enzymatic activities from the different fungi and functional groups differences, elemental composition differences during co-culture with different fungi were performed using SPSS 17.0 software (SPSS, USA).

**Figure 1:**
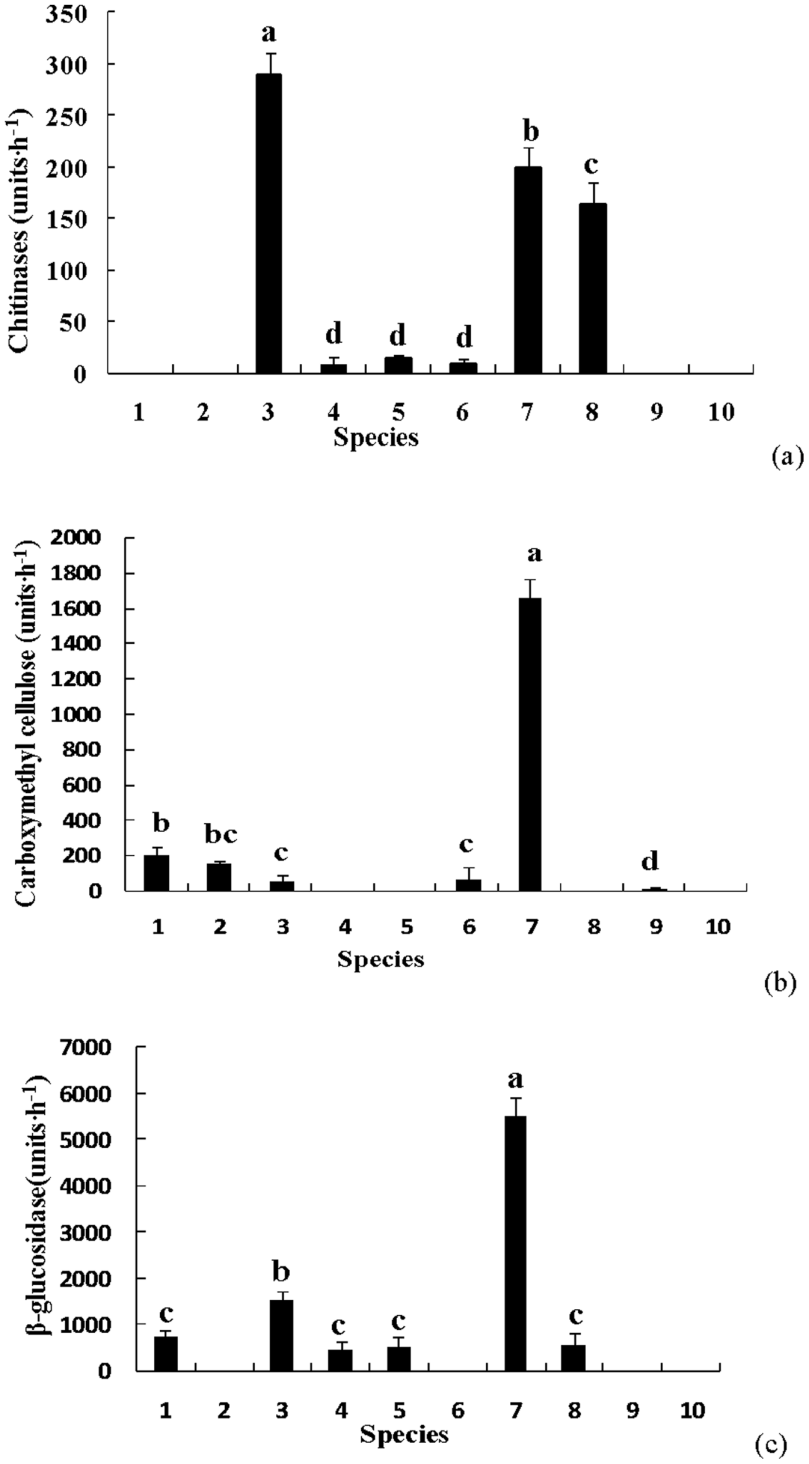
Cellulose and chitin metabolism related enzyme activity. a) chitinase; b) carboxymethyl cellulase; c) β-glucosidase. X axis labels: 1. *R. integra*; 2. *S. granulatus*; 3. *P. impudicus*; 4. *P. adiposa*; 5. *C. dryophila*; 6. *A. sylvicola*; 7. *C. striatus*; 8. *G. rutilus*; 9. *L. deliciosus*; 10. *G. mammosum*

**Figure 2:**
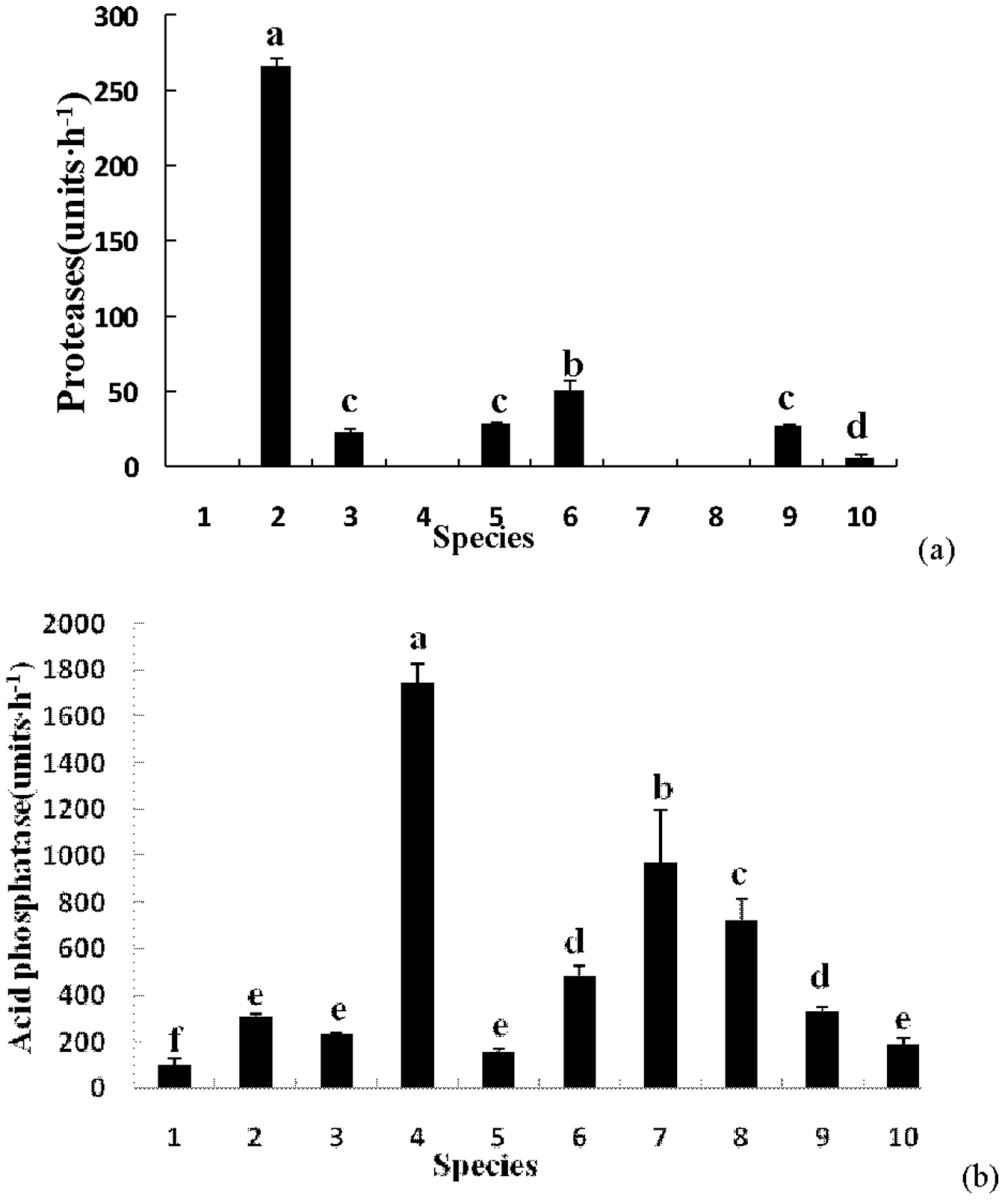
Protein and phosphorus metabolism related enzyme activity. a) protease; b) acid phosphatase X axis labels are the same to Fig. 1.

**Figure 3:**
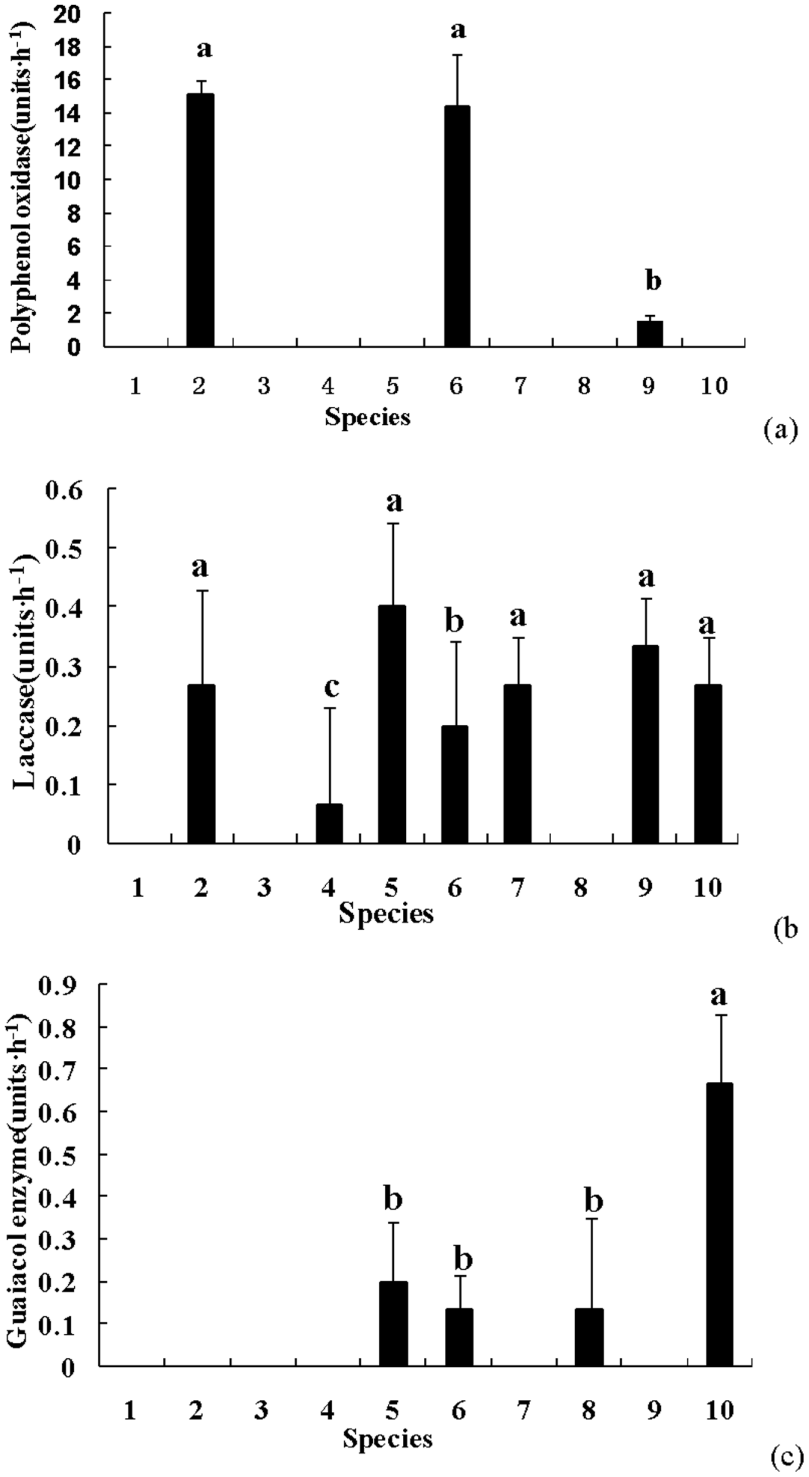
Lignin metabolism related enzyme activity. a) Polyphenol oxidase; b) Laccase; (c) Guaiacol oxidase. X axis labels: 1. *R. integra*; 2. *S. granulatus*; 3. *P. impudicus*; 4. *P. adiposa*; 5. *C. dryophila*; 6. *A. sylvicola*; 7. *C. striatus*; 8. *G. rutilus*; 9. *L. deliciosus*; 10. *G. mammosum*

For making a comprehensive comparison, the fungi with the highest enzymatic activity (those with the significant letter of *a* on the column in Figs. 1, 2 and 3) were marked as 6 plus (++++++). Thereafter, the fungi with the significant letter *b, c, d, e,* and *f* on the column was marked as 5 plus (+++++), 4 plus (++++), 3 plus (+++), 2 plus (++) and 1 plus (+), respectively (as shown in Table 2). In some cases, the higher plus number was marked when two letters in the same column. For example, 5 plus (+++++) was labeled when a column with significance letter of *bc.*

**Table 2:** Comprehensive analysis of eight EEAs secreted by 10 fungi.

No	Species	Chitinase	Carboxymethyl cellulase	β-glucosidase	Protease	Acid phosphatase	Polyphenol oxidase	Laccase	Guaiacol oxidase	Enzymatic types
1	*R. integra*	**-**	**+++++**	**++++**	**-**	**+**	**-**	**-**	**-**	**3**
2	*S. granulatus*	**-**	**+++++**	**-**	**++++++**	**++**	**++++++**	**++++++**	**-**	**5**
3	*P. impudicus*	**++++++**	**++++**	**+++++**	**++++**	**++**	**-**	**-**	**-**	**5**
4	*P. adipose*	**+++**	**-**	**++++**	**-**	**++++++**	**-**	**++++**	**-**	**4**
5	*C. dryophila*	**+++**	**-**	**++++**	**++++**	**++**	**-**	**++++++**	**+++++**	**6**
6	*A. sylvicola*	**+++**	**++++**	**-**	**+++++**	**+++**	**++++++**	**+++++**	**+++++**	**7**
7	*C. striatus*	**+++++**	**++++++**	**++++++**	**-**	**+++++**	**-**	**++++++**	**-**	**5**
8	*G. rutilus*	**++++**	**-**	**++++**	**-**	**++++**	**-**	**-**	**+++++**	**4**
9	*L. deliciosus*	**-**	**+++**	**-**	**++++**	**+++**	**+++++**	**++++++**	**-**	**5**
10	*G. mammosum*	**-**	**-**	**-**	**+++**	**++**	**-**	**++++++**	**++++++**	**4**
Fungus types		**6**	**6**	**6**	**6**	**10**	**3**	**7**	**4**	

+: detectable enzyme, **-**: undetectable enzyme; An increasing number of plus signs indicates an increasing level of enzymatic activity. As shown in Figs.1 to 3, the fungi with the highest enzymatic activity together with those of no significant differences (those with the letter of *a* on the column) was marked as 6 plus (++++++). Thereafter, the fungi with the letter *b*,c,*d*,*e*, and *f* on the column of Figs.1,2 and 3 was marked as 5 plus (+++++),4 plus (++++), 3 plus(+++), 2 plus(++) and 1plus(+), respectively.

## Results

### 1 Enzymes involved in cellulose and chitin metabolism from different fungi

As shown in Fig 1 (a), *P. impudicus*, *C. striatus*, and *G. rutilus* had obviously higher chitinase activity than any of the other species (>163 units·h^−1^). In particular, *P. impudicus* had a peak activity of about 290 units·h^−1^. *C. dryophila*, *A. sylvicola,* and *P. adiposa* had very weak activity, about 8–14 units·h^−1^ (p<0.05). Chitinase activity in *R. integra*, *S. granulatus*, *L. deliciosus*, and *G. mammosum* were undetectable.

Carboxymethyl cellulase and β-glucosidase are two major cellulose decomposition enzymes. As shown in Fig. 1 (b and c), *C. striatus* had much higher carboxymethyl cellulase activity (1659 units·h^−1^) and β-glucosidase activity (5516 units·h^−1^) than other species (p<0.05). Looking at cellulase activity, *R. integra*, *S. granulatus*, *P. impudicus*, and *A. sylvicola* had relatively weak enzymatic activity, about 56–206 units·h^−1^. Minimal enzymatic activity was found in *L. deliciosus* (8.47 units·h^−1^) and activity was undetectable in *P. adiposa*, *C. dryophila*, *G. rutilus*, and *G. mammosum*. In the case of β-glucosidase, enzymatic activity detected from *R. integra*, *C. dryophila*, *G. rutilus*, and *P. adiposa* ranged from 460 to 746 units·h^−1^, but the enzyme was undetectable in *S. granulatus*, *A. sylvicola*, *L. deliciosus*, and *G. mammosum*.

### 2 Enzymes involved in protein and phosphorus metabolism among the different fungi

Two enzymes related to protein and phosphorus metabolism, proteinase and acid phosphatase, were surveyed (Fig. 2). As shown in Fig. 2 (a), *S. granulatus* had the highest proteinase activity than any of the other species (p<0.05), 267 units·h^−1^. *P. impudicus*, *C. dryophila*, *A. sylvicola*, *L. deliciosus*, and *G. mammosum* had relatively low enzymatic activities, 6–51 units·h^−1^. The enzyme was undetectable in *R. integra*, *P. adiposa*, *C. striatus*, and *G. rutilus*.

All fungi could secrete acid phosphatase, but each fungus had different levels of phosphatase activity (Fig. 2 b). The enzymatic activity of *P. adiposa* (1745 units·h^−1^) was highest (p<0.05), *C. striatus* was 973 units·h^−1^, followed by *G. rutilus* at 727 units·h^−1^. Relatively lower enzymatic activity, 105–483 units·h^−1^, were found in *R. integra*, *S. granulatus*, *P. impudicus*, *A. sylvicola*, *L. deliciosus*, and *G. mammosum*.

### 3 Enzymes involved in lignin metabolism among the different fungi

Three types of enzymes, polyphenol oxidase, laccase, and guaiacol oxidase were measured (Fig. 3). As shown in Fig. 3(a), only *S. granulatus, A. sylvicola*, and *L. deliciosus* could produce polyphenol oxidase, while this enzyme in all other fungi was undetectable. *S. granulatus* and *A. sylvicola* had higher enzymatic activities (about 15 units·h^−1^) than *L. deliciosus* (1.47 units·h^−1^) (p<0.05).

As shown in Fig. 3 (b), laccase activity in all fungi was detectable except *R. integra, P. impudicus* and *G. rutilus*. *C. dryophila* had the highest levels of laccase activity, about 0.4 units·h^−1^. This was followed by *L. deliciosus*, *S. granulatus*, *C. striatus*, *G. mammosum* (about 0.20–0.33 units·h^−1^) and they were not different from that of *C. dryophila* (p>0.05). *P. adiposa* had the lowest activity, with only 0.07 units·h^−1^, which was significantly lower than other detectable fungi (p<0.05).

As shown in Fig. 3 (c), 4 out of the 10 fungi (*C. dryophila*, *A. sylvicola*, *G. rutilus*, and *G. mammosum*) had guaiacol oxidase activity, while activity in all other fungi was undetectable. Peak enzymatic activity of 0.67 units·h^−1^ was found in *G. mammosum* (p<0.05), while the guaiacol oxidase activity of *C. dryophila*, *A. sylvicola*, and *G. rutilus* (0.13 to 0.20 units·h^−1^) had non-significant inter-specific differences (p>0.05).

### 4 Enzymatic differences between the fungi and their inter-correlations

Figs. 1 to 3 show the differences in the 8 enzymes among the 10 fungi species. For a comprehensive view of the EEA activity among the different fungi, Table 2 makes an overall comparison of the differences in enzymatic secretion by symbolically representing the statistical differences in Figs. 1 to 3 (as shown in material and method). Some fungi, such as *R. integra*, *P. adiposa*, *G*. *rutilus*, and *G*. *mammosum* could secret 3–4 types of EEAs with relative lower activities. However, some other fungi, such as *S. granulatus, P. impudicus*, *C. dryophila*, *A. sylvicola*, *C. striatus*, and *L. deliciosus* could secrete more than 5 types of EEAs, which usually had high enzymatic activities. Accordingly, we classified these fungi into two groups of high (≥5 types) and low enzyme (≤4 types) producers (Table 2).

### 5 IR Results: Fungi affect the functional group traits on soil colloids

**Figure 4:**
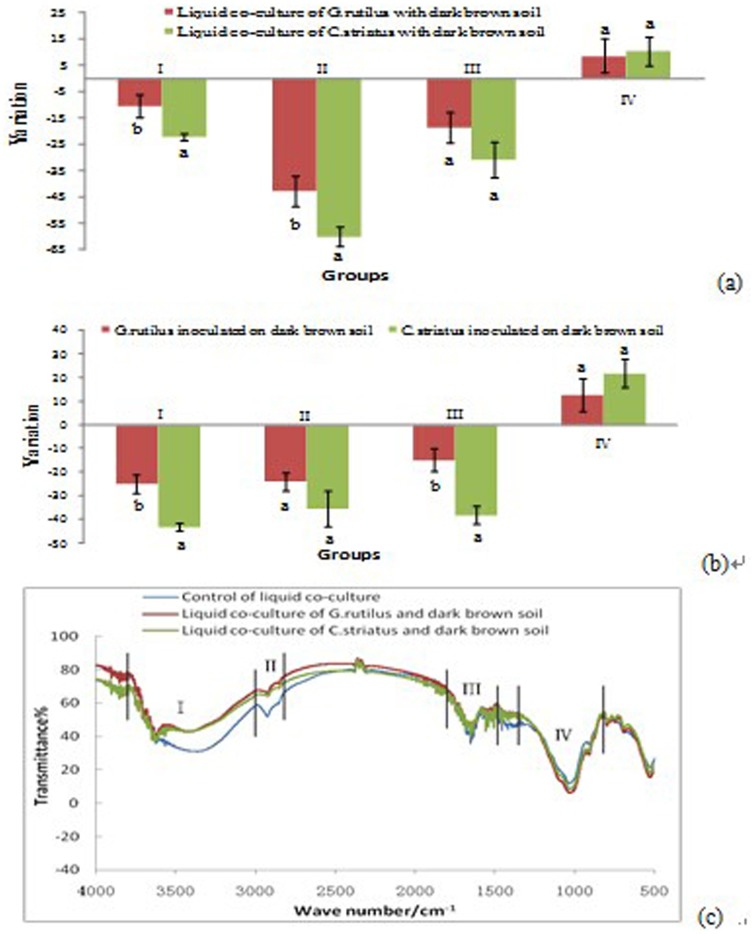
Functional traits changes after liquid (a) and solid (b) co-culture with high enzymatic fungi (*C. striatus*) and low enzymatic fungi (*G. rutilus*). A schematic map is shown in (c) describing the infrared spectrum in liquid co-culture, and the map for solid co-culture is similar. Labels of X axis (I,II,II,IV) can be found in Table 1.

**Figure 5:**
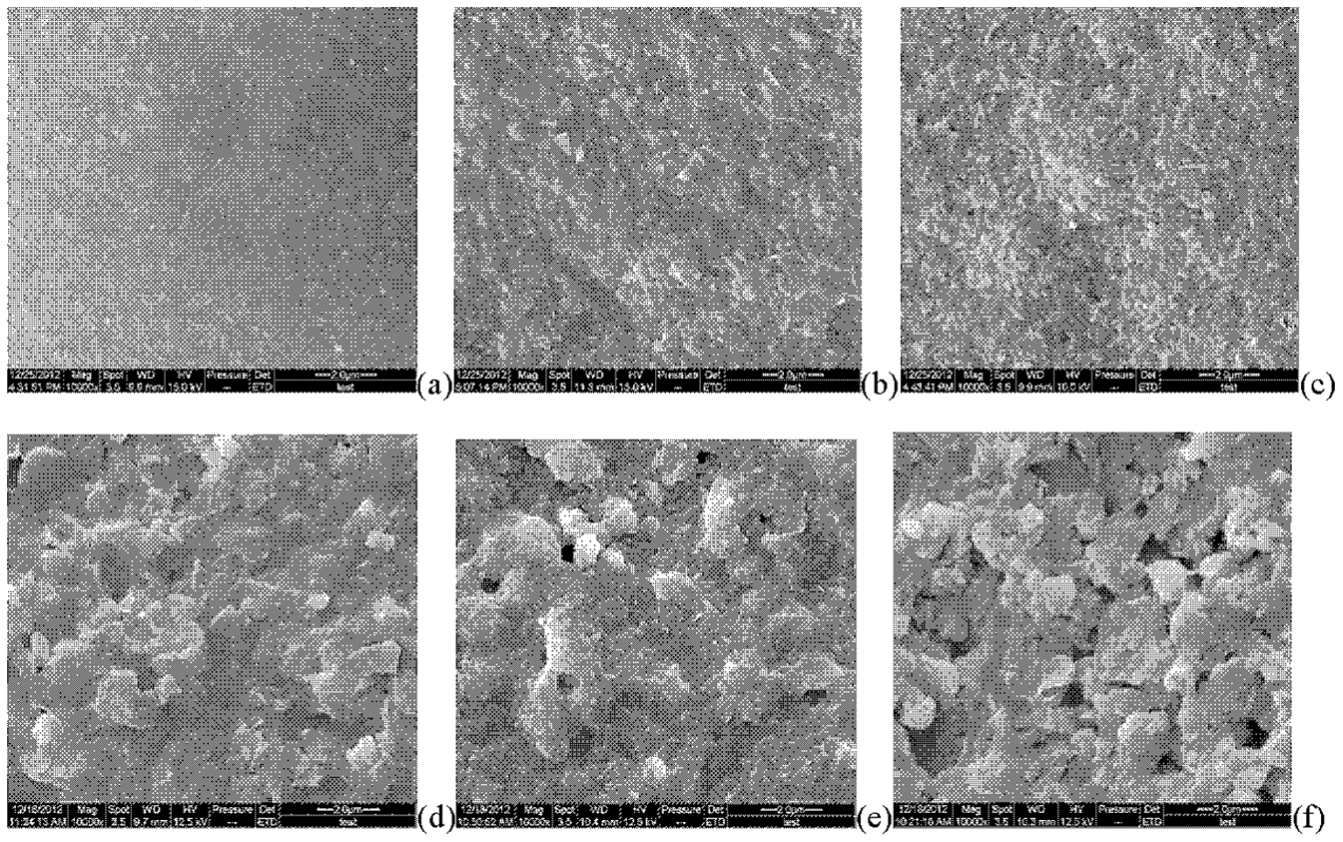
Scanning electron microscopy images of soil colloids after co-culture. The first row is liquid co-culture with two fungi and the second row is solid co-culture with two fungi. Control: a) and d); low enzymatic fungi, *G. rutilus*, b) and e); high enzymatic fungi, *C striatus*, c) and f).

Instead of co-culturing all of the fungi, 1 fungus with high enzymatic activities (*C. striatus*) and one fungus with low enzymatic activities (*G. rutilus*) were co-cultured with dark brown soil for observation of functional group changes (Fig. 4), elemental composition changes (Table 3), and surface image changes (Fig. 5) on soil colloids.

**Table 3:** Elemental composition alterations during liquid and solid co-culture with soil via energy dispersive X-ray microanalysis.

Co-culture types	Treatment	Molecular weight of the element(Wt%)									
		C	N	O	Mg	Al	Si	K	Ca	Fe	Total
Liquid	Control	37.3a	14.7a	23.8a	0.41a	10.0a	7.2a	1.60a	1.16a	3.59a	100
	Low enzymatic fungi of *G. rutilus*	19.3b	14.4a	24.9a	1.37a	11.9a	15.1b	3.72a	2.21a	5.05a	100
	High enzymatic fungi of *C. striatus*	20.7b	14.3a	22.8a	1.02a	12.4a	13.1b	4.04a	1.54a	4.79a	100
Solid	Control	16.4a	14.9a	26.6a	1.01a	11.5a	20.1a	1.63a	1.27a	6.55a	100
	Low enzymatic fungi of *G. rutilus*	16.8a	14.7a	28.1a	1.05a	11.0a	19.7a	1.48a	1.07a	6.18a	100
	High enzymatic fungi of *C. striatus*	14.7b	14.5a	26.0a	0.96a	11.6a	21.3a	1.46a	1.22a	6.25a	100

By adding either of these fungi in solid and liquid co-culture, variable functional groups of I (O-H, N-H stretching,aromatic C-H stretching), II (aliphatic C-H stretching), and III (C = O, asymmetric COO- stretching) decreased 11–60%, while general increases (9–22%) were found in group IV (P = O, C-O stretching, O-H bending and Si-O-Si stretching) (Fig. 4). Compared with the liquid co-culture, solid co-culture of soil and fungi produced more reduction in group I (25–43% vs 11–22%), less reduction in group II (24–35% vs 43–60%), similar changes in group III (15–38% vs 19–31%) and more increase in group IV (13–22% vs 9–10%) (Figs. 4 a & b).

Although the shape of IR spectrum is similar for different fungi and different cultures (Fig. 4c), the size of effects varied between species, i.e. the higher enzymatic activity led to greater changes in functional groups (Figs. 4 a &b). Treatment with *C. striatus* (high enzymatic activity) led to greater changes of most of the functional groups compared to *G. rutilus* (low enzymatic activity). In liquid co-culture, there was a larger reduction in group I, II and III with *C. striatus* (23%, 60%, and 30%, respectively) than in *G. rutilus* (10%, 43%, and 15%, respectively), and similar results were found in the solid co-culture. The increase in group IV after co-culture with *C. striatus* was also larger than that seen with *G. rutilus*, although the difference did not reach significance (p>0.05).

### 6 SEM Results: Fungi affect the surface image of soil colloids

As shown in Fig. 5a, in the liquid co-culture, the organic substance adhere to the surface of soil colloids made the shape of soil particles not obvious in the control compared with that in co-culture with fungi. After co-culture with dark brown soil in liquid medium for 15 days, the edges of the soil colloid became clearly visible, the soil colloid particles became larger and began sticking together, and the differences between two the fungi species were not evident (Fig. 5b,c).

When fungi were inoculated on dark brown soil in solid co-culture, the edges of the soil colloid particles were much smoother, and the small gaps between colloid particles became fuzzy-like in appearance; this gap was invisible in the control (Fig. 5d). When co-cultured, the gaps between the edges of the soil particles became visible. In particular, the co-culture with *C. striatus* (the higher enzymatic producer) induced larger inter-particle spaces (Fig. 5e, f).

### 7 EDX Results: Fungi affect the elemental composition of soil colloids

EDX results are shown in Table 3. *C. striatus* and *G. rutilus* co-cultured with dark brown soil in liquid medium had significantly changed the soil elementary compositions on the surface of the soil colloid. C was reduced from 37% to 20% (p<0.05). Solid co-culture with dark brown soil showed a similar trend, but the magnitude was much smaller. For example, C in the soil colloids decreased from 16.41% to 14.68% in co-culture with the higher enzymatic producer of *C. Striatus* (*p*<0.05), but no marked differences were found between control and the lower enzymatic producer of *G. rutilus*. For all other elements, the changes from co-culture with fungi were not evident (*p*>0.05) (Table 3).

## Discussion

Fungi play an important role in boreal forest ecosystems, both as decomposers of SOM and as root-associated mediators of belowground C transport and nutrient cycling [12], [18], [40]. Ectomycorrhizal fungi and arbuscular mycorrhizal can benefit forest trees by enhancing soil nutrient uptake, particularly for elements with low mobility in the soil such as P and micronutrients [8], [9], [41], [42]. Our result proved that different fungi differed in their enzymatic secretions related to C, N and P metabolism, and these enzymatic differences could possibly affect the surface structure and chemical composition of soil colloids. In the following sections, these findings will be discussed with comparison of previous studies and data in present study.

Different soil fungi may function differently in soil C, N, and P cycling and differences in both the type and activity of the fungal EEA secretions from the present study support this statement (Table 2, Figs. 1–3). Six out of the 10 tested fungi produced EEA related to chitin and cellulose metabolism (chitinase, B-glucosidase, and carboxymethyl cellulase), which directly affect C storage in soil [43]. Similarly, six fungi could participate in soil protein hydrolization, while all fungi could bring about soil phosphorus utilization due to their stable phosphatase activity (Table 2). Lignin hydrolization and decomposition is an important function of fungi in ecosystem nutrient cycling. Three to seven fungi were able to affect lignin decomposition through the secretion of polyphenol oxidase (*S. granulate, A. sylvicola*, and *L. deliciosus*), laccase (*S. granulates*, *P. adiposa*, *C. dryophila*, *A. sylvicola*, *C. striatus*, *L. deliciosus*, and *G. mammosum*), and guaiacol oxidase (*C. dryophila*, *A. sylvicola*, *G. rutilus*, and *G. mammosum*). These enzymes could degrade lignin and various xenobiotic aromatic compounds [12]. Previous studies agree that soil fungi differ in enzymatic profiles, suggesting that these different fungi may play roles at different parts of SOM decomposition, and our data build on these previous studies (Table 2). In a *Quercus petraea* forest, the highest cellulase activities were found in Penicillium strains, and the highest activity of chitinolytic enzymes was found in *Acremonium* sp., while the production of the hemicellulose-degrading enzymes α-galactosidase, β-galactosidase, and α-mannosidase was mostly low for all 29 strains of nonbasidiomycetous microfungi [43]. The successive changes in litter chemistry are reflected in differential enzyme activity and changes in the microbial community composition [44]. Moreover, these microbial interactions including variable arbuscular mycorrhizal fungi can drive ecosystem functions such as plant biodiversity, productivity, and variability [45], and enzymatic differences is considered one driver of these important roles [5], [44].

Enzymatic interactions with soil particles are crucial for enhancing plant nutrient absorption and for promoting the nutrient cycling of SOM. One of our findings is that EEAs secretion can affect functional groups on the surface of soil colloids during co-culture. Moreover, fungi with higher enzymatic activities (*C. striatus*) usually induced larger reduction of functional groups compared with the fungi with lower enzymatic activities (*G. rutilus*) (Fig. 4). Most C- and N-related functional groups traits in the soil colloid were reduced, such as N-H stretching of amines, amides, aromatic C-H stretching, C = O stretching of carboxylic acids, amides, ketones, and COO- stretching of carboxylic acids salts, O-H stretching of carboxylic acids, phenols, alcohols, clay minerals and oxides as well as sorbed water [39]. Enzymatic differences had an effect on organic functional groups traits in soil colloids during the co-culture with fungi (Fig. 4), which may be related to soil colloid nutrients absorption and extracellular enzymatic decomposition in the process of fungal growth [25], [46]–[49]. These enzymatic differences between *C. striatus* and *G. rutilus* both in types and activity (Figs. 1, 2, 3) may play a role in the decomposition processes of macromolecular substances in SOM and the infrared functional traits variation [25], [50], [51]. Chitinase is an important digestive enzyme that can hydrolyze *β*-1,4-glycosidic linkages in chitin, an insoluble linear homopolymer of *β*-1,4-linked N-acetylglucosamine (NAG) residues [52]. Cellulases (such as carboxymethyl cellulase and β-glucosidase) are widely distributed enzymes that hydrolyze the glycosidic bond between two or more carbohydrates or between a carbohydrate and non-carbohydrate moiety [53]. Proteases catalyze amide (peptide) bond hydrolysis in protein or peptide substrates [54]. Laccases use oxygen as the electron acceptor to remove protons from phenolic hydroxyl groups. This reaction gives rise to radicals that can spontaneously rearrange, which can lead to fission of C-C or C-O bonds of the alkyl side chains, or to cleavage of aromatic rings [55]. Polyphenol oxidases catalyze the oxidation of ortho-diphenols to the corresponding quinines [56]. To sum up, fungi-induced reduction in C- and N-related compounds in soil colloids is related with their enzyme secreting differences, and the higher the enzymatic activity the more reductions of C and N functional groups there are.

Beyond functional group traits, the variations of the elementary compositions and surface structure of soil colloids were also affected by fungi-culture with soils and may be regulated by the fungal enzymatic activities (Fig. 5 and Table 3). EEAs secreted by fungi function to decompose organic macromolecular material and create a food supply for fungi growth. EDX results showed C reduced 44–48% after liquid co-culture, while a smaller reduction (11%) was found in solid co-culture with *C. striatus*. SEM images showed that the control soil colloids in liquid medium was attached to many organic substances on the surface of the soil colloid, and co-culture with fungi resulted in a clearly visible surface with soil colloidal particles sticking together (Fig. 5). In the solid co-culture, a similar tendency was also observed. All these changes differed from with the enzymatic profile of the two fungi. High enzymatic activity usually accompanied a high reduction in C and obvious changes in surface image (Fig. 5 and Table 3). Fungi can grow faster in liquid medium with shaker culture than on the dark brown soil and static culture, which made a much clearer observation of soil colloids in liquid co-culture than those in solid co-culture (Table 3 and Fig. 5). When fungi were not inoculated into the soil, SEM images revealed adhesive materials on the surface of soil colloids from dark brown soil. These viscous materials wrapped around soil particles, filled some gaps among particles, and induced smoother surfaces with unclear edges compared with those co-cultured with fungi.

Different from C- and N-related compounds on soil colloids, P-related functional groups (P = O together with C-O stretching, O-H bending and Si-O-Si stretching) were increased 9–22% (Fig. 4). As shown in Table 2, both *C. striatus* and *G. rutilus* had high acid phosphatase. The higher the acid phosphatase activity, the more P = O-related phosphate in soil colloid increased. P limitation for plant growth is well-reported [9], [57], and mycorrhizal infection may improve the absorption of P from soil [8], [58]. Acid phosphatases, which are involved in plant phosphate nutrition, seem to be key enzymes in the utilization of complex phosphate esters, and have been widely studied over the past few years [6], [25]. Acid phosphatase will hydrolyze *p*-nitrophenylphosphate, releasing *p*-nitrophenol and inorganic phosphate (Pi), the only phosphate form taken up by plants and microorganisms [6], [25], [49], [59]. Our results support the idea that a majority of fungi can produce acid phosphatases, increasing the amount of labile Pi for the plant to use, and these should relate with P-related functional traits changes on soil colloids.

## Conclusions

EEA types and activities secreted by different fungi vary, and such enzymatic differences affect functional groups traits, element composition, and surface images of soil colloids when co-cultured with fungi. Some fungi (*R. integra*, *P. adiposa*, *G. rutilus*, and *G. mammosum*) can secrete 3–4 types of enzymes, while others (*S. granulatus, P. impudicus*, *C. dryophila*, *A. sylvicola*, *C. striatus*, and *L. deliciosus*) can secrete more than 5 types of enzymes, all with relatively higher enzymatic activity, showing their possible divergent function in soil C, N, and P cycling. Most functional groups related with C and N were markedly decreased, while increases in P-related functional groups were observed, which may be a basis for the fungi-induced P increases for plant growth. Both SEM and EDX data showed changes on the colloid surface and elemental compositions of the soil colloids. Investigations of this process via soil fungi enzymatic differences and interaction with soil colloids may promote the understanding of the underlying mechanisms driving soil nutrient cycling.
